# Experience-Sampling Methodology with a Mobile Device in Fibromyalgia

**DOI:** 10.1155/2012/162673

**Published:** 2012-12-09

**Authors:** Castilla Diana, Botella Cristina, García-Palacios Azucena, Farfallini Luis, Miralles Ignacio

**Affiliations:** ^1^Labpsitec, Universitat Jaume I, Avenida Sos Baynat s/n, 12071 Castellón de la Plana, Spain; ^2^CIBER Physiopathology of Obesity and Nutrition CIBERobn, CB06/03, Instituto de Salud Carlos III, Spain

## Abstract

This work describes the usability studies conducted in the development of an experience-sampling methodology (ESM) system running in a mobile device. The goal of the system is to improve the accuracy and ecology in gathering daily self-report data in individuals suffering a chronic pain condition, fibromyalgia. The usability studies showed that the developed software to conduct ESM with mobile devices (smartphones, cell phones) can be successfully used by individuals with fibromyalgia of different ages and with low level of expertise in the use of information and communication technologies. 100% of users completed the tasks successfully, although some have completely illiterate. Also there seems to be a clear difference in the way of interaction obtained in the two studies carried out.

## 1. Introduction

Chronic pain is one of the most common causes of disability, affecting millions of people around the world. Fibromyalgia (FM) is a chronic musculoskeletal pain condition with unknown etiology, characterized by widespread pain accompanied by fatigue and disturbed sleep and mood [1]. Patients remain symptomatic and do not improve over long periods of time.

There is a growing interest in the study of fibromyalgia in order to understand the mechanisms underlying this condition and to offer a better treatment response to the individuals who suffer this impairing disease.

In order to achieve this goal it is important to improve the assessment of key variables like pain and fatigue intensity and mood, among others. One methodology that could be very useful in this field is experience-sampling methodology (ESM) aimed to explore the daily experience of individuals suffering different pathologies (in our case fibromyalgia). ESM is a within-day self-assessment technique in which participants are prompted at established or random intervals to report on relevant variables related to their health condition. This method presents several advantages over traditional assessment. Some authors [2–5] highlight the main advantages of ESM, including: (a) enhances ecological validity because it assesses participants in their normal daily environment; (b) minimizes retrospective bias by assessing the participant's experience in the moment; (c) allows for an examination of the context of experiences. Ecological momentary assessment (EMA) is a repeated sampling of behaviors and experiences of the subject in real time and in real environment. EMA reduces the effects of retrospective biases and provides ecological information to a better understanding of the processes of psychiatric or medical disorders [6, 7].

According to Trull and Ebner-Priemer [3], one clinical application of ESM/EMA could be to monitor treatment progress. Also, they state that mobile electronic devices can be used successfully to administer treatments, suggesting that ESM/EMA data can be helpful in clinical assessment, specifically, to provide a detailed account and understanding of an individual's problems as experienced in daily life [3].

In other recent study, focused on exploring prospective associations between daily behavioral anger expression and daily chronic pain intensity, Bruehl and colleges [8] assert that electronic EMA diary methods have numerous advantages over others. Data are less influenced by various potential sources of bias in recalled memory of pain: recency effects, peak experiences, biasing of mood, and the impact of current pain intensity.

Mobile devices are a good means of conducting ESM and one of the research lines of our team is to explore the utility of mobile devices in the psychological assessment and intervention of chronic pain. 

With the evolution of mobile devices, the concept of *context* of use has gained relevance from the usability point of view [9–11]. Mobile devices allow having access to a wide number of situations in which technology was too invasive or inaccessible until recently. Its small size and portability are their main advantages; however, these same features make design and usability an important challenge. First, a small size screen involves not being able to design interactive elements smaller than the fingers, and second, the size limits the number of functions to be implemented [12]. Optimizing the number of steps and functions and the size and colour of the elements constitute important issues in the design process. Fortunately, since the emergent of the first smartphones until now, a wide number of usability guidelines have appeared, showing the growing interest from the human-computer interaction (HCI) field for mobile devices [13–16].

The features of the mobile device, the context of use, and the final user are essential variables to take into consideration in the development of any application. In the case of fibromyalgia sufferers, they are between 40 and 60 years old [17]. Besides, it is important to take into consideration that most of these users are on the other side of the *digital divide* [18] and their difficulties in the use of ITC could be bigger than the ones observed in younger individuals [19].

This work describes the usability study conducted in the development of an application which goal is to adapt three self-report scales to mobile devices. The final goal is to have available an experience-sampling methodology (ESM) for the ecological evaluation of key variables in chronic pain that could be used by individuals of different ages and different level of expertise in the use of ICT (Information and communications technology).

## 2. Method

### 2.1. Professionals Involved in the Design

In order to assure that the objectives of the study were met a multidisciplinary team participated in the usability studies. The team of professionals involved in the design of the application included a clinical psychologist with expertise in the field of chronic pain, a psychologist with expertise in usability, and a computer engineer.

### 2.2. Pilot Study 1: Evaluation of High Fidelity Mockups

The objective of study 1 was to conduct a user test with nonfunctional prototypes in the form of high fidelity mockups.

#### 2.2.1. Initial Specifications

The aim of the software for mobile devices (smartphones) was to gather in real time self-report measures key in chronic pain: intensity of pain, intensity of fatigue, and general mood. Data are daily downloaded to the database automatically.

The self-report scales are perceived pain intensity, perceived fatigue intensity (11-point scales), and general mood (7-point scale, represented with emoticons):

*“Please, indicate the number that best describes the intensity of the pain you are feeling now:”*

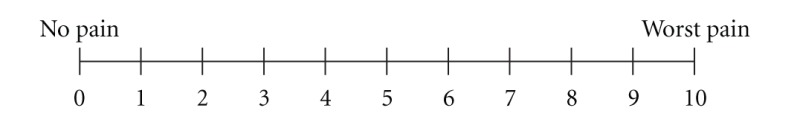

* “Please, indicate the number that best describes the intensity of the fatigue you are feeling now:” *

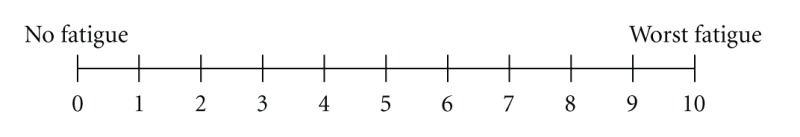

*“Please, indicate the face that best describes your current mood:”*

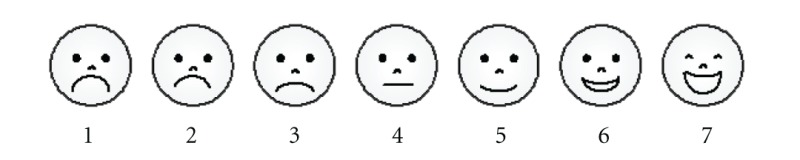


The design should adapt these scales including all the features to a screen of 240 × 320 pixels.

**Figure 1:**
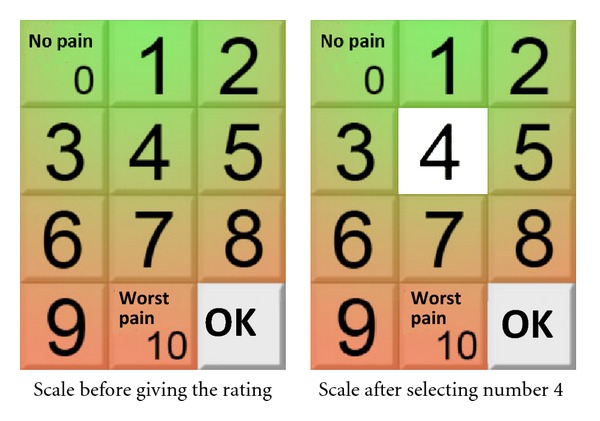
Specification: before pushing the OK button.

Taking into account that the final users are middle age and older, with very low or no expertise in the use of smartphones, it is very likely that they experience a low control over the technology. On the other hand, it is necessary, given the final use of the application, that they are confident in the ratings they give. In order to increase the perceived control over the ratings, one feature included in the three scales was to confirm the ratings. In order to achieve this goal the metaphor of pushing the button was used. In this way the selection of the rating was visible. After this the application required to press an OK button to confirm the selection. In Figure 1, the pain scale is displayed. On the left we offer the image of the scale before the selection of the user, and on the right the image of the scale after selecting number 4. Besides, in order to make easier that the user understand the meaning of the scale the colour of the buttons were graduated from green (less pain or fatigue) to red (more pain or fatigue).

The size of the typography was also considered to make easy reading the numbers and words. 22-point size sans-serif was established for the words and 72-point size for the numbers.

The system gathered the data in the following two ways:

*Predetermined and Automatic. *The assessment will be carried out three times a day. A default scheduled was determined: at 9, 15, and 21 h. It was possible to change these times in order to meet the daily schedules of the different users.

An audio signal will indicate the user that it is the time to give the ratings in the three scales. If the user does not give the ratings the audio signal will sound again every 15 minutes for the first hour and once an hour until 2 hours before the next assessment. After that time, the application will consider that the user is absent and that the assessment has not been performed.

*Free Access. *The users will be able to access the scales when they want. To access the application they just have to touch the screen and confirm the intention of giving ratings by pushing the button: Accept.

#### 2.2.2. Sample

The sample included 4 women who met the American College of Rheumatology (ACR) fibromyalgia criteria [17].  Table 1 summarizes the characteristics of the sample. Their age ranged from 40 to 66 years. Three users had a primary study level and one a secondary school level.

Regarding the technological profile of the participants, 3 different areas could be identified as follows.

- Level of use of computers/Internet/e-mail: frequency of use of each technology.
- Level of use of cell phone: Frequency of use, perceived ease of use, being able to read SMS and being able to write SMS.
- Level of use of touch screen: use of touch screen devices, perceived ease of use.

None of the users had any previous experience with the use of smartphones.

With regard to the technological profile, the sample has a very low level of expertise in the use of computers/Internet/e-mail, an heterogeneous level of expertise in the use of the cell phone (low = use of cell phone just to talk; moderate = use of cell phone to talk and read SMS; high = use of cell phone to talk read and write SMS); and an heterogeneous level of expertise in the use of touch screens.

The participants did not present any cognitive impairment. Their auditory and motor skills were suitable in order to follow a conversation and interact with the touch screen.

#### 2.2.3. Procedure

The participants were referred by the rheumatology unit of the major hospital in the area (Hospital General de Castellon) in order to receive psychological treatment for chronic pain management. All were volunteers and signed an informed consent.

The researchers contacted each participant to set a date in order to conduct the usability study. The study was conducted at the Psychology and Technology Lab (Labpsitec) at Universitat Jaume I in Castellon, Spain.

Each user participated in a session that was recorded in order to make the suitable analysis. The experimental session had an average duration of 30 minutes and the four sessions were conducted in a single day.

The materials used in the test were as follows.

**Figure 2:**
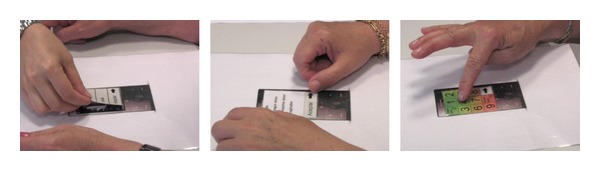
Mockups.

- High fidelity mockups representing the instructions and assessment scales. A picture of the mobile device (real size) was also included in order to facilitate a context of use and to make more understandable the tasks to be performed (see Figure 2).
- Questionnaire designed *AD HOC* for this study.

The participants were told that they will be asked to make a simulation of the assessment of pain intensity, fatigue intensity and general mood using a mobile device with three scales. They were asked to act as if they were alone, encouraging them to say out loud any thoughts regarding the use of the device.

The main evaluation protocol used in this study was the empirical method of task performance measures, obtaining quantitative measures of performance: success/failure rate in each task and time to complete the task. Besides, the participants were asked about the experience using a questionnaire designed for this study. The main objective of the questionnaire was to assess ease of use and the users' opinion regarding the different elements.

#### 2.2.4. Results and Usability Issues Identified

All users interacted with high fidelity mockups using the index finger.

The task of giving ratings in the three scales was successfully performed by all the participants. The opinion regarding the application (represented by high fidelity mockups) was very positive (see Table 2). All participants said the device was easy to use and they consider the design and the size of the elements suitable. Besides, 75% of the users thought they would be able to use the device.

The 75% of the users show some difficulties to understand how to advance. Initially, they did not understand the metaphor of the button, represented by an arrow. Despite this, they learnt fast and the difficulties showed just in the two first screens (out of 12). All participants suggested a change in the design of this element. Some users also suggested that the use of the system involved too many steps.

#### 2.2.5. Recommendations to Improve the Application

After the test, the following recommendations were done to respond to the difficulties found by the participants.

**Figure 3:**
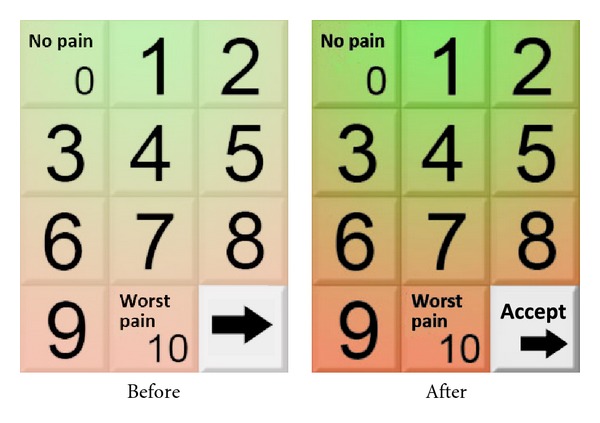
Colour of the scales, before and after performing the evaluation.

- Increase the intensity of the colour in the scales (see Figure 3).
- Change the accept button, including the “Accept” label text.
- Add audio to the written instructions.

All the recommendations were successfully implemented. The result was the development of the software FEMA-M v.1.0 (Fibromyalgia Ecological Momentary Assessment- Mobile v.1.0.). This software was tested in study 2.

### 2.3. Pilot Study 2: First Functional Prototype. User Test

The objective of study 2 is to test the first functional prototype of the software FEMA-M v.1.0.

#### 2.3.1. Sample

The sample included 5 women meeting ACR fibromyalgia criteria [17]. Their age ranged from 36 to 66 years. Only one of the participants had a university degree. The other 4 had a primary study level.

As it can be seen in Table 3 regarding the technological profile of the sample, the level of expertise in the use of computers/Internet/e-mail is very low, the level of expertise in the use of the cell phone is either low or high. Finally, they reported a heterogeneous level of expertise in the use of touch screen.

The participants did not present any cognitive impairment. Their auditory and motor skills were suitable in order to follow a conversation and interact with the touch screen.

#### 2.3.2. Description of the Application

The system is designed to gather self-report information about three key variables in chronic pain: perceived pain intensity (on an 11-point scale), perceived fatigue intensity (on an 11-point scale), and general mood (on a 7-point scale represented with emoticons). The different ratings are represented in 80 × 80 pixel buttons with a gradation in colour from green (for ratings representing less pain and fatigue and positive mood) to red (for ratings representing more intense pain and fatigue and negative mood).

The system includes 12 screens with lineal navigation. The instructions are presented using text accompanied by audio (a male voice).

**Figure 4:**
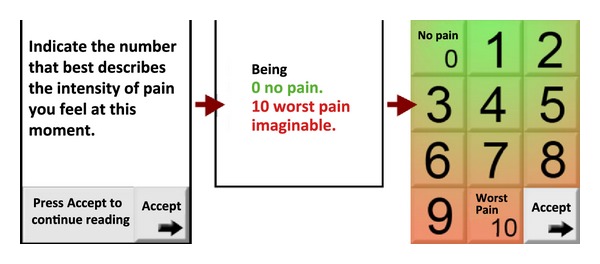
Pain intensity scale.

The chosen typography was 21-point sans-serif in order to facilitate the reading on the screen by the final users (middle age and older individuals). The inconvenient of using this size is that it is necessary to divide the information in several screens (2-3 screens by scale). In Figure 4 an example of the information and the pain intensity scale is displayed. (Figure 4—pain intensity scale).

In order to highlight the visibility of the “Accept” button, the icon of the arrow appears and disappears with a 2 second lapse.

The hardware used was a smartphone HTC Diamond 1: 51 × 102 × 11.5 mm; ROM 4352 MB; RAM 192 MB; 480 × 640 display resolution; 2.8′′ display diagonal; 16 bit/pixel display colour depth; audio stereo sound.

#### 2.3.3. Procedure

The five participants were referred from the rheumatology unit of the major hospital in the area (Hospital General de Castellon) in order to receive psychological treatment for chronic pain management. All were volunteers and signed an informed consent.

The researchers contacted each participant to set a date in order to conduct the usability study. The study was conducted at the Psychology and Technology Lab (Labpsitec) at Universitat Jaume I in Castellon, Spain.

Users were instructed to answer the questions showed by mockups, trying to think that they were in a real situation, interacting with the smartphone alone, and answering the questions posed by the program without the possibility of getting any help during the task.

In this way, if the users asked for help during the task performance, this was rated as not completed or failed.

Each user participated in a session that was recorded in order to make the suitable analysis. The experimental session had an average duration of 35 minutes and the five sessions were conducted in a single day.

The recordings were subsequently reviewed by a usability expert and an engineer.

An example of the system and the evaluation process (study 1 and 2) is offered at: http://www.labpsitec.uji.es/eng/multimedia/videosFM1.php.

#### 2.3.4. Results and Usability Issues Identified

The task: “Give a rating for each of the three questions that the application presents” was successfully completed by all the participants. The participants reported a positive view of the application. Participants reported that:

- the device was easy to use (100% of users),
- they relieve they could use the system at home without help (100% of users),
- they liked the system (100% of users),
- they liked the design of the system (100% of users),
- the size of the elements was adequate (100% of users),
- the typography was readable (100% of users).

Most users (80%) reported that the touch technology was easy to use.

The element of the system that the users disliked most (2 users) was the characteristics of the voice (they did not like the intonation and fact that it was a male voice).

One user said that the system had too many screens, but 80% (4 users) reported that they liked the length and the fact that it is possible to advance at a low pace.

The review of the video recordings showed that 100% of users used the smartphone with one hand, interacting with the thumb. This way of holding the device provoked some difficulties during the interaction. An example of this can be seen in the video aforementioned at the end of the procedure section (from minute 1:45 to minute 2: 06).

## 3. Conclusion

One of the first lessons learned in this work, although it may be obvious, is the valuable contribution of the users to the design of any tool. In our case their participation in the study allows us to develop a suitable design, meeting both the clinical requirements and the needs of the users. One of the main challenges of the design was to strengthen the prevention of response errors. Given that this software is aimed at collecting mental health data, the interaction was guided by the following issues.

- That the user was able to understand completely the instructions to provide a rating to each of the three scales.
- That the user was able to visualize the chosen option and confirm that choice.

In order to implement these goals, several specifications were conducted. First, the size of the text was maximized to ensure the understanding of the instructions. This decision involved that the number of screens to present the 3 scales was 12. Some of the users reported that going through the total system was too long (20%), but, at the same time, most users reported that the rhythm and length of the presentation of the scales was adequate (80% of users). From a clinical point of view, this finding led us to take the decision of maintaining the size of the text despite the fact that going through the system was longer; that is, we sacrificed the comfort of the user in order to ensure the clinical utility of the application.

On the other hand, one of the results of study 2 that we consider relevant was that most users used the thumb to interact with the touch screen. This fact made us think that the use of this finger could increase the likelihood of making mistakes related with the selection of the ratings on the scales. Because of that we added to the design a feature that makes the user confirm the selection of the ratings: the user pushes the selected “button,” this adopts the image of a pushed button, and then the user pushes “Accept” to confirm the selection and continue. This feature was the most difficult to understand for the users. However, although the reported difficulties, a 100% of the users were able to complete the task and the learning curve was so fast (2-3 screens) that the difficulties disappeared very quickly. Our conclusion with regard to this issue was that despite this action decreased the intuitive value of the tool, provoking some puzzlement in the users (who expected that the system reacted automatically when they pushed the button), the learning curve was very fast and the benefits that this feature added to the application increased its utility and accuracy, given that the user could select an option and change it before confirm that choice (see Figure 3).

The design of the elements (metaphors, colours, size of the text, etc.) and the choice of the text labels are particularly important when the space is tight. However, the findings from study 1 and 2 related to the “Accept” button indicated that it was preferable to sacrifice some size in the text of the button and maintain the label to guide the users.

One of the issues we would like to emphasize is that although both studies had positive results in the resolution of tasks (a 100% success rate in both studies), we observed a clear difference in the way of interaction observed in each study. In study 1, conducted with high fidelity mockups, 100% of users interacted with the index finger. The mockups, displayed in paper, were placed on a table. However, in study 2, users took the device in their hand, and interacted with the thumb of the same hand. The graphical user interface (GUI) in both cases was the same, however using hardware support added value to the assessment of the GUI, since users changed their behavior when presented the GUI in a smartphone. This hardware influence in the interaction, and taking in to account the variety of types and sizes of smartphones available today, makes us reflect about the need of using definitive hardware models in the usability evaluation, even in early development stages.

Another issue that it is worth mentioning is that, despite the users quickly learnt to use the smartphone, a clear learning curve existed. Whereupon, giving these devices to end users, it may be beneficial to include a brief period of training with a professional (e.g., 10 minutes).

In a future line of work, we want to compare the way users interact with mobile devices of different sizes, and observe how they pick up the device, which hand they use to interact with the device, if they use only one hand or both, and the acceptability of the software depending on the hardware selected.

## Supplementary Material

This video illustrates the FEMA-M system user evaluation during the study: first usability evaluation using HI-FI paper mock-up, and the second using HI-Fi Prototype with full functionality.

## Figures and Tables

**Table 1:** Study 1: Sample description.

ID	Gender	Age	Level of education	Level of expertise computers/internet/e-mail	Level of expertise cell phone	Level of expertise touch screen
User 1	Female	66	Primary study level	Low	Moderate	Moderate
User 2	Female	40	primary study level	None	Moderate	Moderate
User 3	Female	64	Primary study level	None	Low	Low
User 4	Female	55	Secondary school level	High	High	None

**Table 2:** Users' opinion (Mockups).

Questions	Answers user study 1
Do you think the device was easy to use?	Yes 100%
Would you change anything?	The accept button (100%)
Do you think the size of the elements was adequate?	Yes (100%)
Do you like the design?	Yes (100%)
Do you think you would be able to use this device at home without help?	Yes (75%)

**Table 3:** Study 2: Sample description.

User number	Gender	Age	Education	Level of expertise computers/internet/e-mail	Level of expertise cell phone	Level of expertise touch screen
User 1	Female	65	Primary study level	High	High	High
User 2	Female	64	Primary study level	None	High	Moderate
User 3	Female	40	Primary study level	None	Low	Low
User 4	Female	66	Primary study level	None	Low	Low
User 5	Female	36	University degree	None	High	High
